# Early time-restricted carbohydrate consumption vs conventional dieting in type 2 diabetes: a randomised controlled trial

**DOI:** 10.1007/s00125-023-06045-9

**Published:** 2023-11-16

**Authors:** Domenico Tricò, Maria Chiara Masoni, Simona Baldi, Noemi Cimbalo, Luca Sacchetta, Maria Tiziana Scozzaro, Giulia Nesti, Alessandro Mengozzi, Lorenzo Nesti, Martina Chiriacò, Andrea Natali

**Affiliations:** 1 Department of Clinical and Experimental Medicine, University of Pisa, Pisa, Italy; 2 Laboratory of Metabolism, Nutrition, and Atherosclerosis, University of Pisa, Pisa, Italy; 3 Interdepartmental Research Center Nutrafood ‘Nutraceuticals and Food for Health’, University of Pisa, Pisa, Italy; 4 Institute of Life Science, Sant’Anna School of Advanced Studies, Pisa, Italy; 5 Center for Translational and Experimental Cardiology (CTEC), Department of Cardiology, University Hospital Zurich, University of Zurich, Zurich, Switzerland

**Keywords:** Blood glucose control, Circadian rhythm, Dietary carbohydrates, Glucagon, Glucose-dependent insulinotropic peptide, Nutrition therapy, Obesity, Randomised controlled trial, Time-restricted eating, Type 2 diabetes

## Abstract

**Aims/hypothesis:**

Early time-restricted carbohydrate consumption (eTRC) is a novel dietary strategy that involves restricting carbohydrate-rich food intake to the morning and early afternoon to align with circadian variations in glucose tolerance. We examined the efficacy, feasibility and safety of eTRC in individuals with type 2 diabetes under free-living conditions.

**Methods:**

In this randomised, parallel-arm, open label, controlled trial, participants with type 2 diabetes and overweight/obesity (age 67.2±7.9 years, 47.8% women, BMI 29.4±3.7 kg/m^2^, HbA_1c_ 49±5 mmol/mol [6.6±0.5%]) were randomised, using computer-generated random numbers, to a 12 week eTRC diet or a Mediterranean-style control diet with matched energy restriction and macronutrient distribution (50% carbohydrate, 30% fat and 20% protein). The primary outcome was the between-group difference in HbA_1c_ at 12 weeks. Body composition, 14 day flash glucose monitoring and food diary analysis were performed every 4 weeks. Mixed meal tolerance tests with mathematical beta cell function modelling were performed at baseline and after 12 weeks.

**Results:**

Twelve (85.7%) participants in the eTRC arm and 11 (84.6%) participants in the control arm completed the study, achieving similar reductions in body weight and fat mass. The two groups experienced comparable improvements in HbA_1c_ (−3 [−6, −0.3] mmol/mol vs −4 [−6, −2] mmol/mol, corresponding to −0.2 [−0.5, 0]% and −0.3 [−0.5, −0.1]%, respectively, *p*=0.386), fasting plasma glucose, flash glucose monitoring-derived glucose variability and mixed meal tolerance test-derived glucose tolerance, insulin resistance, insulin clearance and plasma glucagon levels, without changes in model-derived beta cell function parameters, glucagon-like peptide-1, glucose-dependent insulinotropic polypeptide and non-esterified fatty acid levels. The two diets similarly reduced liver function markers and triglyceride levels, being neutral on other cardiometabolic and safety variables. In exploratory analyses, diet-induced changes in body weight and glucometabolic variables were not related to the timing of carbohydrate intake.

**Conclusions/interpretation:**

The proposed eTRC diet provides a feasible and effective alternative option for glucose and body weight management in individuals with type 2 diabetes, with no additional metabolic benefits compared with conventional dieting.

**Trial registration:**

ClinicalTrials.gov NCT05713058

**Funding:**

This study was supported by the European Society for Clinical Nutrition and Metabolism (ESPEN) and the Italian Society of Diabetology (SID).

**Graphical Abstract:**

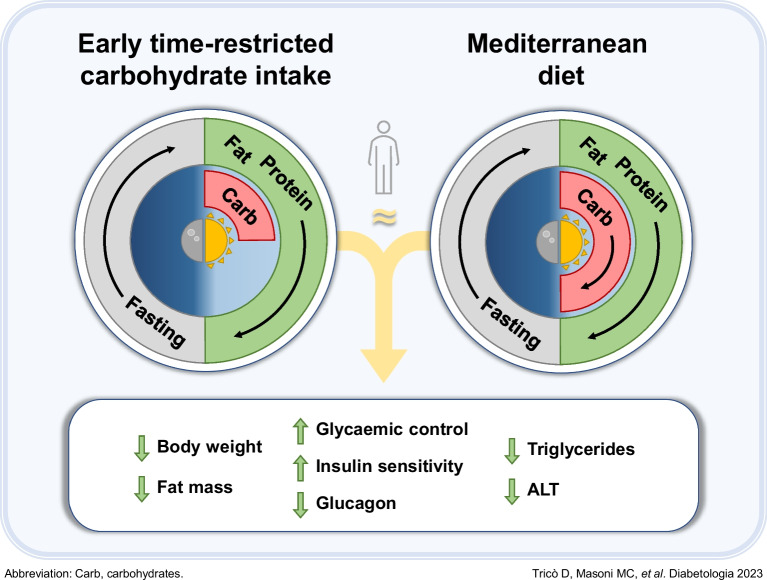

**Supplementary Information:**

The online version contains peer-reviewed but unedited supplementary material available at 10.1007/s00125-023-06045-9.


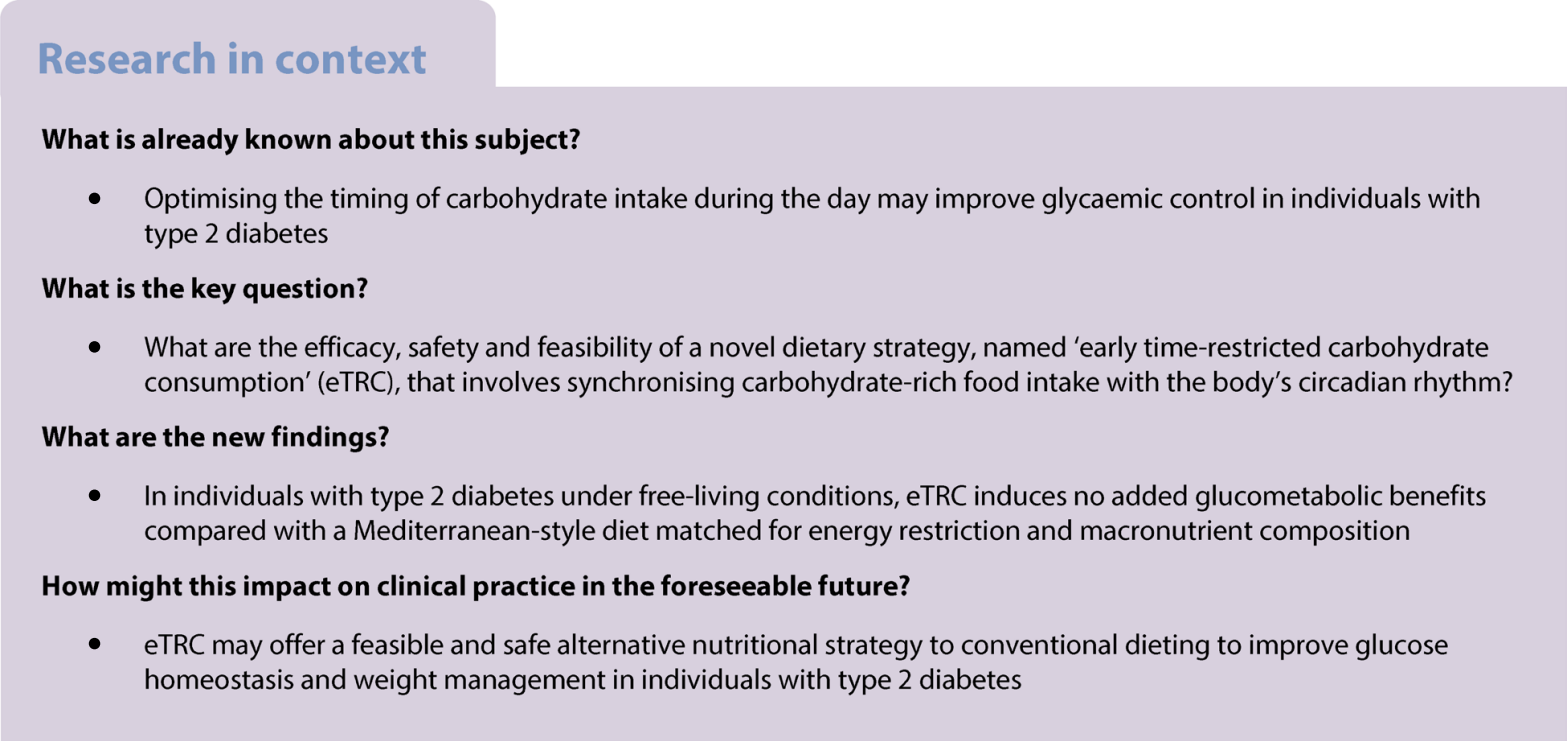


## Introduction

Medical nutrition therapy is essential in the management of type 2 diabetes to improve metabolic and body weight control, reduce the risk of chronic complications and increase both life expectancy and quality [1, 2]. However, controversies still exist regarding the best approach to improve the suboptimal long-term feasibility and cost-efficacy of current dietary strategies, which limit their implementation in clinical practice [3].

In recent years, early time-restricted feeding (eTRF) has emerged as a promising alternative to conventional dieting for its proposed metabolic benefits against the main drivers of glucose dysregulation, encompassing both insulin resistance and beta cell dysfunction [4]. eTRF involves restricting the daily eating window to a limited time frame in the morning and early afternoon, with or without energy restriction. Aligning carbohydrate-rich food consumption to daily variations in oral glucose tolerance [5], which are induced by the body’s circadian rhythm [6], may enhance metabolic flexibility and insulin sensitivity, regardless of body weight loss [4, 6]. Conversely, restricting the eating window to the evening (‘late’ time-restricted feeding [TRF]) had neutral or negative effects on postprandial glucose levels and beta cell responses [7–9]. Furthermore, prolonging overnight fasting intervals up to 18 h promotes ketosis and may result in sustained improvements in cardiometabolic health (up to 12 weeks), similar to those observed with other intermittent fasting schedules [10]. Previous studies on the metabolic benefits of TRF in type 2 diabetes have been limited by the confounding effect of the spontaneous caloric restriction and greater weight loss associated with TRF compared with the control diet [11, 12]. Therefore, whether eTRF improves glucose control and insulin sensitivity in individuals with type 2 diabetes independently of energy intake and weight loss is yet to be established. Moreover, implementing eTRF as a long-term therapeutic strategy poses unsolved challenges, in that the duration of absolute fasting may be impractical and poorly accepted by many individuals, hindering adherence and efficacy.

To address these challenges, we designed a novel alternative dietary strategy, named ‘early time-restricted carbohydrate consumption’ (eTRC). This innovative approach involves restricting carbohydrate-rich food intake to the morning and early afternoon hours to align with circadian variations in oral glucose tolerance, while allowing for a more flexible eating window throughout the day for lipids and proteins to facilitate adherence. This study aimed to provide insights into the feasibility, efficacy and safety of eTRC in people with type 2 diabetes in free-living conditions, focusing on its impact on glucose homeostasis, as compared with a traditional Mediterranean-style diet matched for energy restriction and macronutrient content.

## Methods

### Participants

This trial was conducted at the Section of Clinical Dietology of the University Hospital of Pisa between March 2019 and April 2023. Individuals of either sex (self-reported) between 18 and 75 years of age, regardless of their racial/ethnic background or socioeconomic status, were eligible for the study if their BMI was higher than 25.0 kg/m^2^ and stable for at least 6 months (±0.5 kg/m^2^), they had a clinical diagnosis of type 2 diabetes [13] and they had HbA_1c_ 44–64 mmol/mol (6.2–8.0%) under chronic pharmacological treatment with metformin and/or dipeptidyl peptidase-4 inhibitors only. Individuals were excluded if they were taking other glucose-lowering or weight loss medications or regularly skipped meals. Eligible individuals fulfilling key inclusion and exclusion criteria were invited to participate in this trial during routine outpatient visits. Interested participants were screened to assess full inclusion and exclusion criteria, collect blood samples for routine biochemical analysis and complete medical history and physical examination.

The study protocol was finalised and approved by the local Ethics Committee before the trial was started, while registration at ClinicalTrials.gov (registration no. NCT05713058) was delayed for confidentiality reasons. Informed written consent was obtained from all participants before enrolment.

### Experimental protocol

**Fig. 1:**
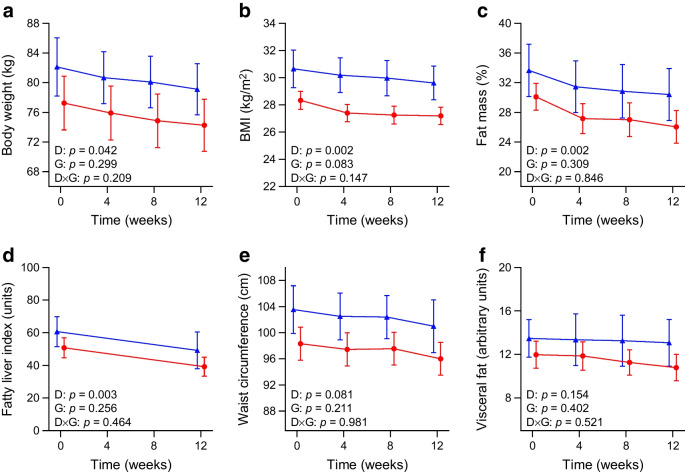
Changes in body weight (**a**), BMI (**b**), fat mass (**c**), fatty liver index (**d**), waist circumference (**e**) and visceral fat (**f**) in individuals with type 2 diabetes randomly assigned to a 12 week eTRC diet (red circles) or a Med diet (blue triangles) with matched energy restriction and macronutrient distribution. Data are mean ± SEM. Group differences were tested by two-way ANOVA for repeated measures including diet (D), group (G) and diet × group (D×G) interaction as factors

The study was conducted as a randomised, parallel-arm, open label, controlled clinical trial, according to the study protocol shown in electronic supplementary material (ESM) Fig. 1. Participants were randomly assigned, in a 1:1 ratio, to follow either an eTRC diet or a Mediterranean-style (Med) control diet matched in energy restriction and macronutrient composition for 12 consecutive weeks. Group allocation was established using computer-generated random numbers. Personnel performing laboratory measurements and mathematical modelling of data were blinded to group allocations. Glucose-lowering medications were kept stable during the trial. Participants were scheduled to attend a total of four visits (one visit per month) with a registered dietitian from randomisation to study end to evaluate changes in body weight and composition, diet adherence and free-living glucose control assessed by 14 day flash glucose monitoring. At weeks 0 and 12, participants underwent a mixed meal tolerance test (MMTT), and fasting blood and urine samples were collected for metabolic and safety assessments.

### Dietary interventions

Both study arms were prescribed a 12 week diet with matched energy restriction (calculated on the individual energy requirement to achieve a 5% body weight loss at 12 weeks using the validated Body Weight Planner available at http://bwsimulator.niddk.nih.gov), macronutrient composition (50% carbohydrates, 30% fat and 20% protein) and meal frequency (3 meals/day). Energy requirements were calculated as the sum of the resting energy expenditure by the Harris–Benedict formula and the estimated energy expenditure during physical activity. Both diets followed the principles of the healthy Mediterranean-style diet pyramid, being rich in whole grain cereals, fruits, vegetables, nuts and seeds, legumes, fish, eggs, poultry and olive oil, and low in red and processed meat, sugar, salt, saturated fat and alcohol [14, 15]. Participants received written dietary guidelines detailing weighted raw foods to be consumed each day, providing a list of suitable self-selected substitutions with similar energy and macronutrient content. Participants randomised to eTRC were instructed to restrict the intake of carbohydrate-containing food to the first two meals of the day (breakfast and lunch), while participants in the Med group were instructed to distribute carbohydrates across the three main meals.

### Compliance assessment

Participants were instructed to complete 3 day quantitative food diaries, with food type and weight, cooking method, condiments used and meal timing, every other week of the trial. At each visit, food diaries were handed to study dietitians for compliance monitoring and assessment of the followed diet characteristics. Energy intake, macronutrient content and daily distribution of carbohydrates were determined independently by two investigators using the Italian Food Composition Tables of the Council for Agricultural Research and Economics. If estimations differed by ≥20%, a third investigator repeated the assessment to resolve any dispute.

### Body composition

Body weight and composition, including visceral fat, were measured in the fasting state by bioelectrical impedance with a Tanita MC780 instrument (Tokyo, Japan). Waist circumference was measured at the midpoint between the lower rib margin and the superior iliac crest.

### Flash glucose monitoring

After randomisation, participants received three FreeStyle Libre 2 sensors (Abbott, Chicago, IL, USA), allowing the quantification of mean glucose, glucose CV (%) and percentage of time spent with interstitial glucose levels below 3.9 mmol/l (<70 mg/dl, time below range), within 3.9 and 10 mmol/l (70 to 180 mg/dl, time in range), above 10 mmol/l (>180 mg/dl, time above range) and above 7.8 mmol/l (>140 mg/dl) or 6.7 mmol/l (>120 mg/dl).

### MMTT

An MMTT was performed at baseline (week 0) and at the end of the diet (week 12). Participants were admitted to the Laboratory of Metabolism, Nutrition, and Atherosclerosis at the University of Pisa after a standardised pre-test evening meal and 12 h overnight fast. A polypropylene cannula was inserted into an antecubital vein and two baseline blood samples were collected. Then, participants were asked to consume a mixed liquid meal (125 ml of Nutridrink Compact, neutral taste, Danone Nutricia, Milano, Italy), consisting of 37 g of carbohydrates (including 19 g of sugar), 12 g of fat and 12 g of protein (total energy 1255 kJ, of which 49.3% was from carbohydrates, 38.7% from fat and 16.0% from protein). Blood samples were collected at 15, 30, 45, 60, 90, 120, 150 and 180 min after meal consumption.

### Blood pressure and heart rate

Blood pressure and heart rate were assessed after 5 min of resting as the mean value of the last two of three consecutive measurements, performed with a validated automated device (Omron M6 Comfort, Kyoto, Japan).

### Blood analyses

Routine biochemical analyses were performed at the clinical laboratory of the University Hospital of Pisa.

Plasma glucose was measured immediately during the MMTT by a GM9 Glucose Analyser (Analox Instruments, Stourbridge, UK). Plasma insulin, C-peptide, NEFA, glucagon, glucagon-like peptide-1 (GLP-1) and glucose-dependent insulinotropic polypeptide (GIP) were measured in a single assay at the completion of the study at the Laboratory of Nutrition, Metabolism, and Atherosclerosis of the University of Pisa. Insulin and C-peptide measurements were performed by electrochemiluminescence on a COBAS e411 instrument (Roche, Indianapolis, IN, USA). Plasma NEFA levels were assayed by standard spectrophotometric methods (Synchron UniCel DxC 600, Beckman Instruments, Fullerton, CA, USA). Glucagon was measured by ELISA (Mercodia, Uppsala, Sweden). GLP-1 and GIP were measured by ELISA (Merck, Darmstadt, Germany).

### Calculations and mathematical modelling

Insulin secretion rate (ISR) was estimated via C-peptide deconvolution [16]. Parameters of beta cell function were calculated by modelling insulin secretion and glucose concentration during the MMTT, as previously reported [17, 18]. This model describes insulin secretion as the sum of two components. The first component is described by the slope and intercept of the quasi-linear dose–response function linking insulin secretion and absolute glucose concentration, termed beta cell glucose sensitivity and ISR at 5.5 mmol/l glucose (ISR@5.5), respectively. This component is modulated by a time-dependent factor termed potentiation that continuously increases during the MMTT. The ratio of potentiation factor values at 160–180 min vs 0–20 min is used to express this potentiation effect. The second component represents the dependence of ISR on the rate of change of glucose concentration and is named beta cell rate sensitivity.

Fasting and total insulin clearance were calculated as the ratios between fasting levels or AUCs of ISR and plasma insulin during the MMTT, respectively [19].

Fasting and MMTT-derived insulin sensitivity were estimated using the HOMA-IR index or the Matsuda index [20], respectively. The MMTT-derived Matsuda index has not been validated against gold-standard techniques in this experimental setting and, therefore, it was used solely for comparative purposes between and within study groups.

The fatty liver index was calculated as *e*^*y*^/(1+*e*^*y*^)×100, where *y*=0.953×log_*e*_(triglycerides)+0.139×BMI+0.718×log_*e*_(γ-glutamyl transferase [GGT])+0.053×waist circumference−15.745 [21].

### Statistical analysis

The primary outcome measure was between-group change in HbA_1c_ at 12 weeks. Thus, a sample size of 22 participants (*n*=11 each group) was calculated to provide at least 80% power to detect a between-group difference of 4 mmol/mol (0.3%) in HbA_1c_ reduction at 12 weeks, deemed clinically significant, with a two-sided non-parametric test, assuming an SD of 3 mmol/mol (0.3%). A total of 27 participants were randomised, allowing for a 20% dropout rate.

Continuous variables with normal and non-normal distribution are presented as mean ± SD or median and quartiles (Q_1_, Q_3_), respectively. Differences between groups were tested by Fisher’s exact test or Mann–Whitney *U* test, as appropriate. Repeated measures were analysed by two-way ANOVA to examine the effects of diet (D), group (G) and diet × group interaction (D×G). For measures repeated during the MMTT, time (0 to 180 min) and time × diet and/or group interaction factors were included as covariates. Correlations were tested by Spearman rank correlation. Statistical analysis was performed based on the initial arm assignment using a complete case analysis. Exploratory analysis was performed by stratifying participants into tertiles of percentage intake of carbohydrates after lunch. Analyses were performed using JMP Pro software version 17.0.0 (SAS Institute, Cary, NC, USA). All tests were conducted at a two-sided α level of 0.05.

## Results

### Study population

The CONSORT study flow diagram is shown in ESM Fig. 2. Among 30 individuals invited to participate in this clinical trial, 27 participants were screened and randomly assigned into the intervention group (eTRC diet, *n*=14) or the control group (Med diet, *n*=13). During the trial, two participants in each group withdrew their consent from the study for lack of motivation, while 12 (85.7%) participants in the eTRC group and 11 (84.6%) participants in the Med diet group completed the protocol and were included in the final analysis (age 67.2±7.9 years, 47.8% women, 100% non-Hispanic white ethnicity, BMI 29.4±3.7 kg/m^2^, HbA_1c_ 49±5 mmol/mol or 6.6±4%). Demographic and metabolic characteristics of the two groups, including age, sex distribution, BMI, body composition, glycaemic control and pharmacological treatment, were not statistically different (Table 1).

**Table 1:** Characteristics of study participants

Characteristic	eTRC diet (*n*=12)	Med diet (*n*=11)	*p*
Age, years	71.5 [59.5, 74.0]	67.0 [63.0, 71.0]	0.688
Women, *n* (%)	5 (41.7)	6 (54.5)	0.684
BMI, kg/m^2^	28.2 [26.6, 29.4]	30.4 [27, 32.8]	0.196
Body weight, kg	76.9 [67.5, 86.5]	82.4 [71.1, 94.5]	0.295
Waist circumference, cm	97.5 [93.3, 101.8]	99.0 [96.0, 110.0]	0.281
Fat mass, %	27.8 [25.3, 35.8]	34.8 [22.7, 44.9]	0.389
Visceral fat, arbitrary units	11.5 [8.3, 15.0]	12.0 [9.0, 17.0]	0.621
Systolic blood pressure, mmHg	139 [128, 149]	130 [119, 157]	0.786
Diastolic blood pressure, mmHg	85 [78, 93]	80 [78, 86]	0.327
Heart rate, bpm	75 [67, 76]	85 [59, 87]	0.461
Diabetes duration, years	6.0 [2.0, 10.0]	5.0 [2.0, 8.5]	0.730
Alcohol consumption, *n* (%)			0.510
Never	10 (83.3)	9 (81.8)	
Often	0 (0)	1 (9.1)	
Frequently	2 (16.7)	1 (9.1)	
Tobacco smoking, *n* (%)			0.507
Never	6 (50.0)	6 (54.5)	
Active smokers	0 (0)	1 (9.1)	
Ex smokers	6 (50.0)	4 (36.4)	
Fasting plasma glucose, mmol/l	7.8 [6.7, 8.0]	7.3 [6.6, 8.9]	0.844
HbA_1c_, mmol/mol	49 [44, 51]	48 [47, 51]	0.755
HbA_1c_, %	6.6 [6.2, 6.8]	6.5 [6.5, 6.8]	0.755
Fasting plasma insulin, pmol/l	95 [44, 111]	86 [56, 171]	0.512
Total cholesterol, mmol/l	4.5 [3.5, 5.0]	4.3 [3.8, 5.3]	0.712
HDL-cholesterol, mmol/l	1.4 [1.3, 1.7]	1.5 [1.2, 1.6]	0.902
LDL-cholesterol, mmol/l	2.7 [1.9, 3.5]	2.6 [2.1, 3.6]	0.622
Triglycerides, mmol/l	1.2 [0.8, 1.7]	1.2 [0.9, 1.6]	0.902
AST, IU/l	17 [16, 20]	22 [19, 27]	0.016
ALT, IU/l	18 [13, 20]	23 [20, 38]	0.011
Creatinine, µmol/l	70 [59, 80]	72 [60, 76]	0.782
Glucose-lowering medications, *n* (%)			
Metformin	7 (58.3)	8 (72.7)	0.469
DPP-4 inhibitors	0 (0)	2 (18.2)	0.122

Data are count (%) or median [Q_1_, Q_3_]

Group differences were tested by Fisher’s exact test or Mann–Whitney *U* test, respectively

DPP-4, dipeptidyl peptidase-4

### Diet composition

The prescribed and reported diet compositions evaluated through food diary analysis are presented in ESM Table 1. As per protocol, the two diets were matched for energy content and macronutrient composition, while carbohydrate intake was differently distributed throughout the day. Compared with the Med group, the eTRC group consumed 58% more carbohydrates at breakfast, similar amounts of carbohydrates for lunch and 69% less carbohydrates after lunch (15.8 [6.3, 23.0] g vs 47.4 [39.7, 61.9] g, *p*=0.0004; mean difference −34.4±6.4 g). The small amount of carbohydrates consumed after lunch by the eTRC group mainly derived from fruit and non-starchy vegetables at dinner.

### Body weight and composition

During the diet, the eTRC and Med diet groups experienced significant and similar reductions in body weight and BMI, consistent with the prescribed daily energy restriction, as well as similar reductions in fat mass and fatty liver index (Fig. 1a–d). Numerical reductions in waist circumference and visceral fat also occurred, albeit not statistically significant (Fig. 1e,f).

### Free-living glycaemic control

**Fig. 2:**
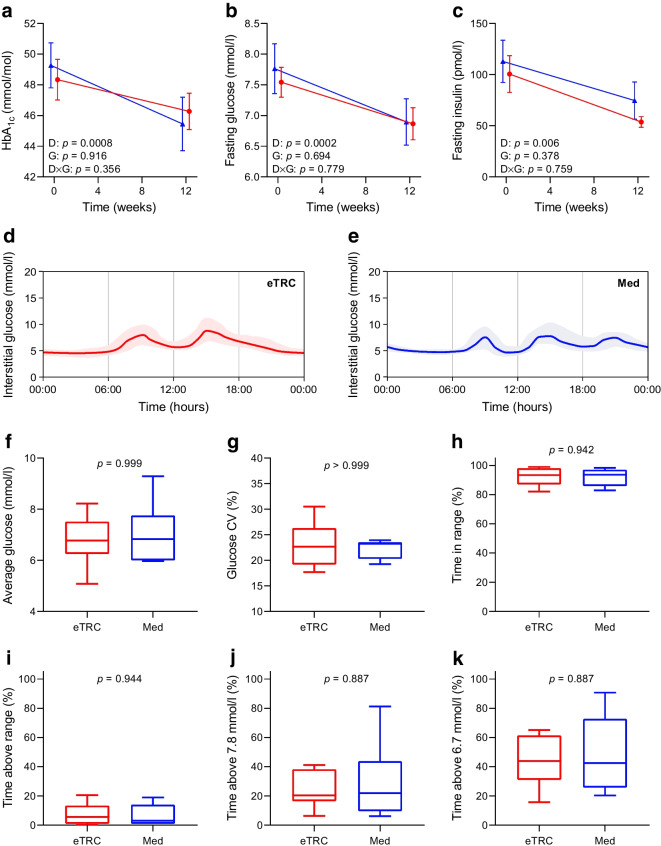
Changes in HbA_1c_ (**a**), fasting glucose (**b**) and insulin (**c**) in individuals with type 2 diabetes randomly assigned to a 12 week eTRC diet (red circles) or a Med diet (blue triangles) with matched energy restriction and macronutrient distribution. Data are mean ± SEM. Group differences were tested by two-way ANOVA for repeated measures including diet (D), group (G) and diet × group (D×G) interaction as factors. Representative 14 day flash glucose monitoring reports of participants from the eTRC diet (**d**) and Med diet (**e**) groups. Median (continuous line) and 10th and 90th percentiles (shaded area) are shown. Flash glucose monitoring-derived average interstitial glucose (**f**), glucose CV (**g**) and percentage of time spent within the target glucose range of 3.9 to 10 mmol/l (**h**), above the target glucose range (**i**) or with interstitial glucose levels above 7.8 mmol/l (**j**) or 6.7 mmol/l (**k**) in free-living conditions. Group differences were tested by Mann–Whitney *U* test

HbA_1c_, fasting plasma glucose and insulin markedly decreased during the trial in all participants, without significant differences between the eTRC and Med diet groups (change in HbA_1c_ −3 [−6, −0.3] mmol/mol and −4 [−6, −2] mmol/mol, respectively, corresponding to −0.2 [−0.5, 0]% and −0.3 [−0.5, −0.1]%, *p*=0.386) (Fig. 2a–c). Inspection of 14 day average glucose profiles in free-living conditions confirmed a different daily distribution of carbohydrate intake in the two groups, showing only two early postprandial glucose peaks in eTRC participants (i.e. breakfast and lunch) and three postprandial glucose peaks in the Med diet group (i.e. breakfast, lunch and dinner) (Fig. 2d,e). Average interstitial glucose, glucose variability, time in range, time above range and time below range recorded by flash glucose monitoring were similar between the two groups (Fig. 2f–i, ESM Fig. 3). Moreover, there were no group differences in the percentage of time spent with interstitial glucose levels above 7.8 mmol/l or 6.7 mmol/l (Fig. 2j,k).

### MMTT

**Fig. 3:**
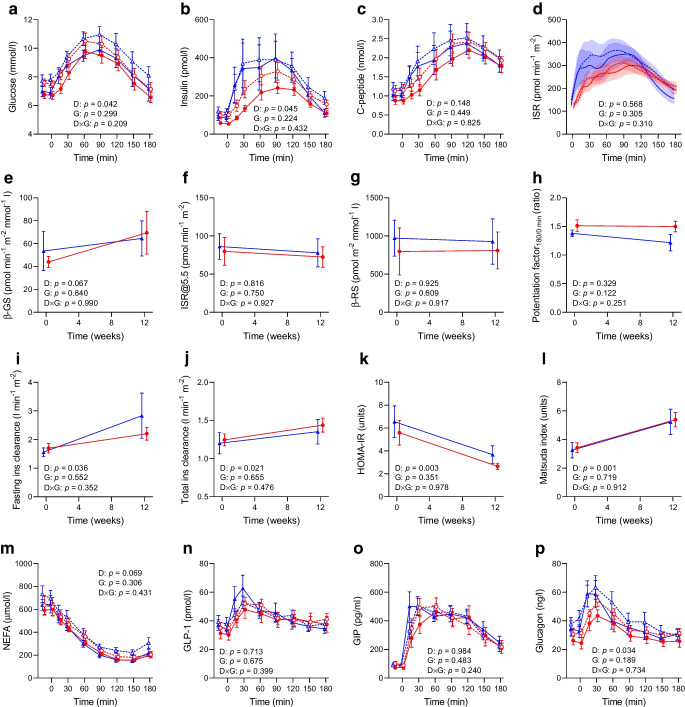
Changes in plasma glucose (**a**), insulin (**b**), C-peptide (**c**), ISR (**d**), model-derived beta cell glucose sensitivity (β-GS) (**e**), ISR@5.5 (**f**), beta cell rate sensitivity (β-RS) (**g**), potentiation factor ratio (**h**), fasting insulin clearance (**i**), total insulin clearance (**j**), HOMA-IR (**k**), Matsuda index (**l**), NEFA (**m**), GLP-1 (**n**), GIP (**o**) and glucagon (**p**) measured during a 180 min MMTT in individuals with type 2 diabetes randomly assigned to a 12 week eTRC diet (red circles) or a Med control diet (blue triangles) with matched energy restriction and macronutrient distribution. Data are mean ± SEM. Dashed lines in panels (**a**–**d**) and (**m**–**p**) indicate baseline values at week 0. Group differences were tested by two-way ANOVA for repeated measures including diet (D), group (G) and diet × group (D×G) interaction as factors. Ins, insulin

Plasma glucose and insulin levels during the MMTT were comparable between groups at baseline and were reduced to the same extent by the diet (Fig. 3a,b). Plasma C-peptide levels, C-peptide-derived insulin secretion and model-derived beta cell function parameters were similar between groups and not significantly affected by the diet, although beta cell glucose sensitivity numerically increased at 12 weeks (diet effect, *p*=0.067) (Fig. 3c–h). Fasting and post-load insulin clearance were similarly increased by the diet in both groups, as well as insulin sensitivity measured by both HOMA-IR and Matsuda index (Fig. 3i–l), while NEFA levels were numerically reduced during the MMTT (diet effect, *p*=0.069) (Fig. 3m). Plasma glucagon levels in response to the mixed meal markedly decreased after the diet, while there were no changes in fasting and post-load GLP-1 or GIP (Fig. 3n–p).

### Other metabolic and haemodynamic measures

Circulating triglyceride levels significantly decreased in both groups after the diet (eTRC: −0.21 [−0.51, 0.15] mmol/l; Med: −0.26 [−0.41, 0.17] mmol/l, diet effect, *p*=0.032; interaction effect, *p*=0.917) and there was a downsloping trend in LDL-cholesterol (diet effect, *p*=0.072), while total cholesterol and HDL-cholesterol were unaffected (ESM Fig. 4a–d). Among liver enzymes, alanine aminotransferase (ALT) (eTRC: −3 [−8, 0] IU/l; Med: −6 [−12, 1] IU/l, diet effect, *p*=0.003; interaction effect, *p*=0.335) and GGT levels (eTRC: −3 [−4, 1] IU/l; Med: −2 [−23, 0] IU/l, diet effect, *p*=0.013; interaction effect, *p*=0.347) were similarly reduced after the diet in the two groups, while aspartate aminotransferase (AST) was unaffected (ESM Fig. 4e–g). The thyroid stimulating hormone was numerically reduced by the diet in both groups (diet effect, *p*=0.054) (ESM Fig. 4h), while no significant changes occurred in kidney function, uric acid levels, erythrocyte sedimentation rate (ESR), blood pressure and heart rate (ESM Fig. 4i–p).

### Exploratory analyses

Exploratory analyses were performed to examine any possible relationships between the actual amount of carbohydrates consumed in the later hours of the day, regardless of the assigned intervention, and the observed changes induced by the diet. In correlation analysis, late carbohydrate intake, expressed as median percentage intake of carbohydrates after lunch, was not associated with changes in body weight, fat mass, fasting glucose, HbA_1c_, insulin clearance, insulin sensitivity and plasma levels of triglycerides and ALT (ESM Fig. 5). Additionally, the effects of the diet were compared between the two extreme tertiles of the distribution of percentage carbohydrate intake after lunch, not differing for main baseline characteristics (*n*=8 each group). Participants belonging to the first tertile consumed almost four times less carbohydrates after lunch (8.0 [4.1, 8.9]%) than the third tertile (30.5 [28.6, 36.4]%, *p*=0.0008), but experienced similar reductions in body weight, fat mass, fasting glucose, HbA_1c_, insulin clearance, insulin sensitivity and plasma triglycerides and ALT (ESM Fig. 6).

## Discussion

This randomised controlled trial tested efficacy, feasibility and safety of a novel dietary strategy, named eTRC, which involves restricting carbohydrate-rich food intake to the morning and early afternoon to align carbohydrate consumption with the body’s circadian rhythm. In people with well-controlled type 2 diabetes under free-living conditions, eTRC induced no added glucometabolic benefits compared with a traditional Mediterranean-style diet matched for energy restriction and macronutrient composition. Furthermore, eTRC appeared to be as effective as conventional dieting for the management of multiple cardiometabolic risk factors, encompassing overweight, increased whole-body and visceral adiposity, insulin resistance, impaired insulin clearance and dyslipidaemia. These beneficial effects appear to be largely conveyed by body weight loss, which intrinsically improves metabolic health, and not related to the daily distribution of carbohydrate intake.

The accurate matching of energy and nutrient prescription between the eTRC and Med diets, as well as the quantification of carbohydrate intake across the day, allowed us to dissect the effects deriving from the specific timing of carbohydrate consumption from those of body weight loss or diet composition. By study design, the two groups randomised to the eTRC or Med diet achieved equal improvements in body weight and composition after 12 weeks of intervention, in line with the prescribed energy restriction. This finding does not support additional benefits of eTRC on weight management as compared with conventional dieting. Consistently, even a stricter regimen of energy-restricted eTRF was not superior to energy restriction alone in reducing body weight and fat mass over a 12 month period [22].

Previous acute studies under controlled conditions suggest that altering the daily distribution of carbohydrate intake can affect 24 h glycaemic control in people with type 2 diabetes [23–25]. Indeed, shifting carbohydrate (86 g vs 14 g) and energy intakes (2946 kJ vs 858 kJ) from dinner to breakfast acutely reduced daily glucose excursions, with the greatest improvements occurring in the evening hours [24]. Building upon this evidence, we sought to examine whether aligning carbohydrate consumption to circadian rhythms and unloading the beta cell during night-time would translate into sustained glycometabolic benefits in individuals with type 2 diabetes, independent of weight loss. Both dietary interventions achieved a significant amelioration of multiple metabolic variables in a short time frame, proving feasible and effective. Nonetheless, the eTRC group experienced no added improvement in fasting blood glucose, insulin and HBA_1c_ levels; real-life glycaemic control assessed by flash glucose monitoring; and glucose tolerance and insulin sensitivity during MMTT. Moreover, there were no detectable differences in fasting and glucose-stimulated insulin secretion assessed by C-peptide deconvolution and model-derived parameters of beta cell function. Neutral effects of the eTRC diet on glucose homeostasis, independent of weight loss, are unlikely to be explained by an incomplete adherence to the prescribed eTRC regimen, given that late carbohydrate consumption was threefold lower in the eTRC group compared with the Med group, frequently consisted of low-glycaemic-index food only (i.e. vegetables and fruits) and was not associated with metabolic outcomes in exploratory analyses. Moreover, although the amount of carbohydrates shifted from dinner to breakfast in the eTRC group was relatively small (~30–40 g), it was still able to elicit evening postprandial hyperglycaemia in the Med group. In contrast, postprandial glucose (and possibly insulin) excursions at bedtime were almost abolished by the eTRC regimen, as demonstrated by flash glucose monitoring profiles, due to the combined effects of minimising carbohydrate intake and largely increasing the relative protein and fat content of the dinner [26].

Supervised controlled feeding trials to test the metabolic effects of eTRF independent of body weight changes have shown large improvements in insulin sensitivity and beta cell responsiveness [4] and reduced 24 h glucose levels [27] in overweight individuals at risk of diabetes. While promising, these results derive from short-term studies that applied prolonged absolute fasting periods in highly controlled settings [4], whose implementation into daily life can be challenging. To date, only a few studies have explored the metabolic effects of eTRF in free-living individuals with type 2 diabetes. Compared with habitual eating, eTRF without energy restriction was previously associated with improved glucose homeostasis and, at least in some instances, positive effects on insulin sensitivity, which may be explained by unintentional weight reduction [11, 12, 28]. In fact, when body weight loss was carefully balanced between interventions, energy-restricted intermittent eTRF was superior to continuous caloric restriction only in improving postprandial glucose responses under controlled conditions (MMTT), with no detectable differences in fasting glucose and insulin levels, HbA_1c_, and markers of insulin sensitivity or beta cell function, largely in agreement with our findings [29].

Nevertheless, we demonstrated several beneficial changes after the dietary intervention. Measuring postprandial responses to a mixed-nutrient meal provides a more comprehensive assessment of glucose homeostatic mechanisms than simple fasting assessments, allowing the exploration of changes in the physiological gut hormone responses to food intake. In our study, plasma glucagon levels in response to the MMTT were significantly reduced by the diet, regardless of daily carbohydrate distribution. This finding is noteworthy as hyperglucagonaemia is a hallmark of type 2 diabetes, whose resolution has been proposed as a key mechanism of diabetes remission [30]. In contrast, GLP-1 and GIP levels were not affected by the diet, either at fasting or during the MMTT, in line with previous observations [28]. Furthermore, several metabolic risk factors improved during the 12 week trial, along with glucose levels. Circulating triglycerides and liver enzymes were markedly reduced by the two interventions, while insulin clearance increased, likely owing to improvements in insulin resistance and intrahepatic fat. All these metabolic alterations contribute to the pathogenesis of diabetes [31–34], and their prompt improvement following weight loss underscores the importance of medical nutrition therapy in the management of diabetes, supported by current guidelines [1]. Previous studies reported heterogeneous actions of eTRF on lipid metabolism, which might be explained by the different study protocols, participants’ characteristics and achieved weight loss [35]. Again, our data suggest that weight loss and improved insulin sensitivity induced by energy restriction most likely account for the observed positive metabolic effects, which do not appear related to the intrinsic characteristics of the diet.

This proof-of-concept study expands the current knowledge on the promising metabolic effects of time-restricted carbohydrate eating in individuals with type 2 diabetes under free-living conditions. However, some limitations should be acknowledged. First, our sample size was relatively small. This was in line with similar pilot studies and the statistical power was adequate to identify large and clinically meaningful effects on body weight and glucometabolic variables. Women and men were equally represented in the two study groups. However, the small sample size did not allow for subgroup analyses stratified by sex, which may reveal sex-related differences in the glucometabolic effects of eTRC. Second, adherence to the diet was evaluated from self-reported 3 day food diaries, which are prone to error and bias. To mitigate the risk of misinterpretations, food diaries were analysed independently by two investigators, and a third investigator was involved to resolve any dispute. Furthermore, flash glucose monitoring allowed the detection of any unprescribed carbohydrate assumption to confirm the strict adherence to the protocol during recording. Predicted and actual weight loss were also very similar, in the absence of significant changes in physical activity and sedentary behaviours, indicating adequate adherence at least to dietary energy prescriptions. Third, dedicated studies should be performed to assess the effects of a longer-term eTRC dietary intervention, compared with both conventional dieting and a more stringent eTRF. Finally, the therapeutic potential of eTRC should be tested in people with less controlled type 2 diabetes, greater habitual carbohydrate intake in the evening or different racial/ethnic background, who may achieve greater metabolic benefits from eTRC.

In conclusion, this randomised controlled trial demonstrated that synchronising carbohydrate intake with the daily variations in glucose tolerance, while allowing for habitual fat and protein eating, does not amplify the glycometabolic benefits of an energy-matched Mediterranean diet in individuals with type 2 diabetes. Nonetheless, tailoring the dietary intervention to the individual’s preferences and habits promotes greater compliance and increases efficacy. Thus, this dietary regimen may offer a feasible and safe alternative nutritional strategy to conventional dieting to improve cardiometabolic risk management in type 2 diabetes.

### Supplementary Information

Below is the link to the electronic supplementary material.

Supplementary file1 (PDF 340 KB)

## Data Availability

Data generated in this study are available from the corresponding authors upon request.

## Abbreviations

ALT: Alanine aminotransferase
AST: Aspartate aminotransferase
eTRC: Early time-restricted carbohydrate consumption
eTRF: Early time-restricted feeding
GGT: γ-Glutamyl transferase
GIP: Glucose-dependent insulinotropic polypeptide
GLP-1: Glucagon-like peptide-1
ISR: Insulin secretion rate
ISR@5.5: ISR at 5.5 mmol/l glucose
Med: Mediterranean-style
MMTT: Mixed meal tolerance test
TRF: Time-restricted feeding
