# Supplementary material for: Early time-restricted carbohydrate consumption vs conventional dieting in type 2 diabetes: a randomised controlled trial
Source: Diabetologia. 2023 Nov 16;67(2):263–74. doi: 10.1007/s00125-023-06045-9 (PMC10789836; doi:10.1007/s00125-023-06045-9)
Supplement: Supplementary file 1 — Supplementary file1 (PDF 340 KB) [file 125_2023_6045_MOESM1_ESM.pdf]

*Electronic supplementary material*

# Early time-restricted carbohydrate consumption versus conventional dieting in type 2 diabetes: a randomized controlled trial

Domenico Tricò[1,2,3*], Maria Chiara Masoni[1,2,*], Simona Baldi[1,2], Noemi Cimbalo[1,2], Luca Sacchetta[1,2], Tiziana Scozzaro[1,2], Giulia Nesti[2], Alessandro Mengozzi[1,4,5], Lorenzo Nesti[1,2], Martina Chiariacò[1,2,4], Andrea Natali[1,2,3]

1. Department of Clinical and Experimental Medicine, University of Pisa, Pisa, Italy
2. Laboratory of Metabolism, Nutrition, and Atherosclerosis, University of Pisa, Pisa, Italy
3. Interdepartmental Research Center Nutrafood "Nutraceuticals and Food for Health", University of Pisa, Pisa, Italy
4. Institute of Life Science, Sant'Anna School of Advanced Studies, Pisa, Italy
5. Center for Translational and Experimental Cardiology (CTEC), Department of Cardiology, University Hospital Zurich, University of Zurich, Zurich, Switzerland

* Equally contributed to this manuscript.

**Correspondence:**

- Domenico Trico, Department of Clinical and Experimental Medicine, University of Pisa, Via Roma 67, Pisa 56126, Italy, Email: domenico.trico@unipi.it
- Andrea Natali, Department of Clinical and Experimental Medicine, University of Pisa, Via Roma 67, Pisa 56126, Italy, Email: andrea.natali@unipi.it

**ESM Tab. 1** – Prescribed and self-reported dietary intake.

| | **eTRC diet** (n=12) | **Med diet** (n=11) | ***p*** |
|---|---|---|---|
| **Prescribed dietary intake** | | | |
| Total Energy, kJ | 6439 [5439, 7360] | 6778 [5812, 7272] | 0.878 |
| Total Energy, kcal | 1539 [1300, 1759] | 1620 [1389, 1738] | 0.878 |
| Carbohydrates, % | 49.0 [48.3, 49.8] | 50.0 [48.0, 50.0] | 0.329 |
| Fat, % | 28.0 [26.3, 30.0] | 29.0 [28.0, 30.0] | 0.609 |
| Protein, % | 23.0 [21.0, 24.8] | 22.0 [20.0, 22.0] | 0.287 |
| **Reported dietary intake** | | | |
| Total Energy, kJ | 5171 [4703, 5406] | 5243 [5067, 7448] | 0.196 |
| Total Energy, kcal | 1236 [1124, 1292] | 1253 [1211, 1780] | 0.196 |
| Carbohydrates, % | 44.5 [36.6, 52.1] | 44.8 [38.2, 51.4] | 0.644 |
| Breakfast | 25.7 [21.2, 33.3] | 16.3 [11.4, 23.7] | 0.008 |
| Lunch | 61.7 [51.1, 67.6] | 57.7 [45.5, 62.6] | 0.325 |
| After lunch | 8.8 [6.4, 15.6] | 28.1 [24.0, 31.1] | 0.0026 |
| Fat, % | 32.0 [27.9, 35.3] | 29.9 [27.5, 35.6] | 0.644 |
| Protein, % | 22.3 [18.9, 26.5] | 21.4 [18.5, 23.4] | 0.460 |

Data are median [$Q_1$, $Q_3$].

**ESM Fig. 1** – Study protocol.

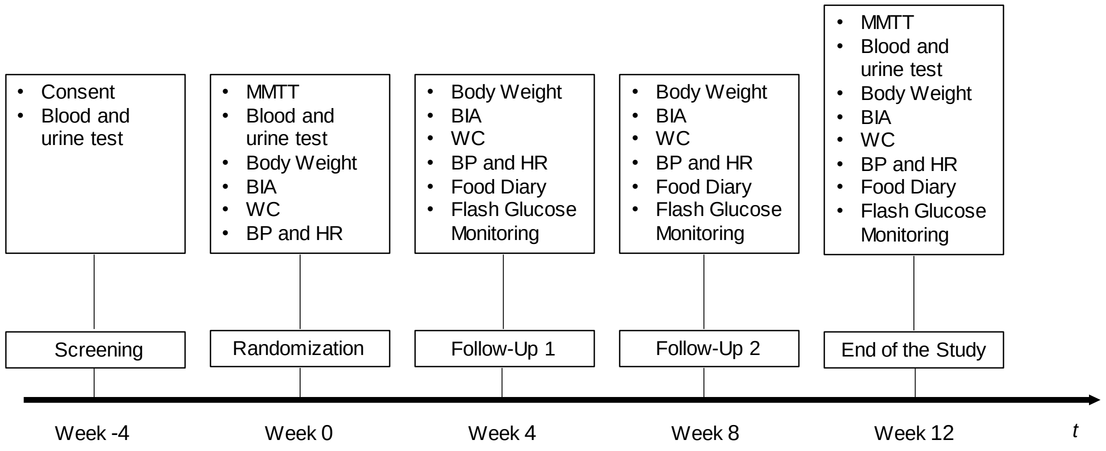


Abbreviations: BIA, bioimpedance analysis; BP, blood pressure; HR, heart rate; MMTT, mixed meal tolerance test; WC, waist circumference.

**ESM Fig. 2** – Study flow diagram.

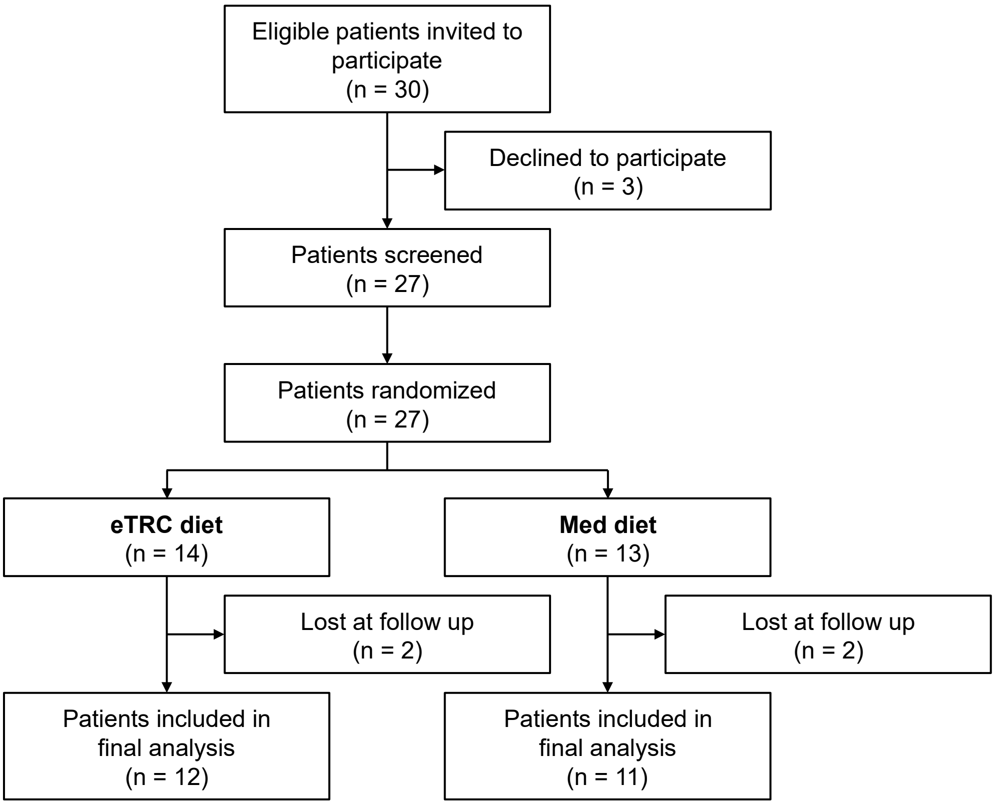

**ESM Fig. 3** – Percentage of time spent with interstitial glucose levels below 3.9 mmol/L (<70 mg/dL).

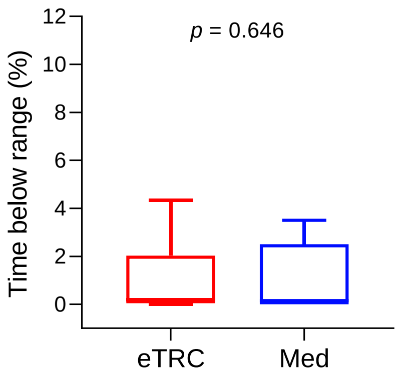


Group differences were tested by Mann–Whitney U test.

**ESM Fig. 4** – Changes in total and fractional cholesterol (**a-c**), triglycerides (**d**), liver markers (**e-g**), TSH (**h**), kidney markers (**i-j**), uric acid (**k**), erythrocyte sedimentation rate (ESR) (**l**), systolic, diastolic, and mean blood pressure (BP) (**m-o**), and heart rate (**p**) in individuals with type 2 diabetes randomly assigned to a 12-week eTRC diet (red circles) or a Mediterranean-style control diet (blue triangles) with matched calorie restriction and macronutrient distribution.

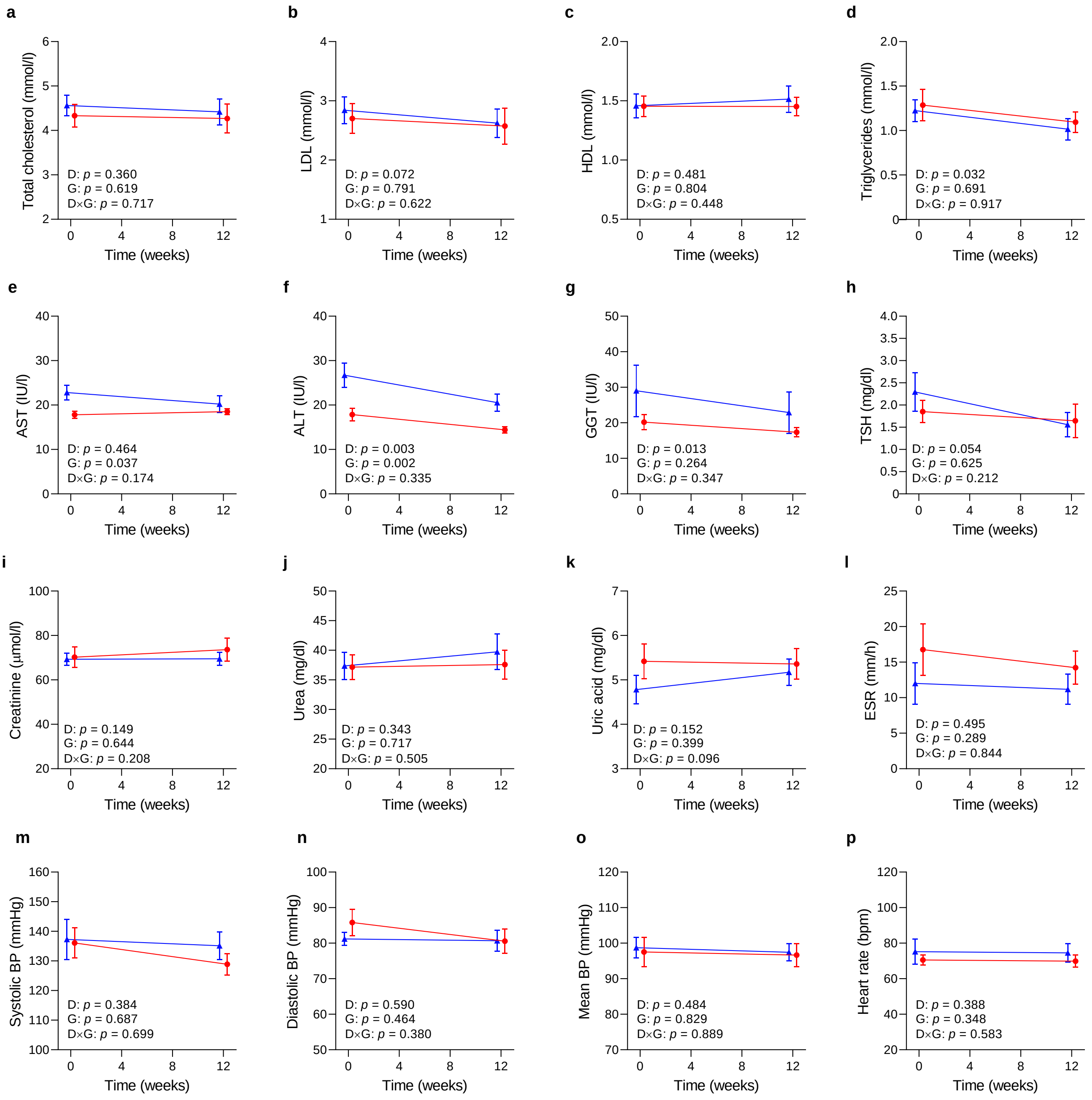


Data are mean ± SEM. Group differences were tested by two-way ANOVA for repeated measures including diet (D), group (G), and diet × group (D×G) interaction as factors.

**ESM Fig. 5** – Correlations between percent carbohydrate intake after lunch during the 12week intervention and diet-induced changes in body weight (**a**), fat mass (**b**), $HbA_{1c}$ (**c**), fasting glucose (**d**), insulin clearance (**e**), Matsuda index (**f**), triglycerides (**g**), and ALT (**h**) in individuals with type 2 diabetes randomly assigned to a 12-week eTRC diet (red circles) or a Mediterranean-style control diet (blue triangles) with matched calorie restriction and macronutrient distribution.

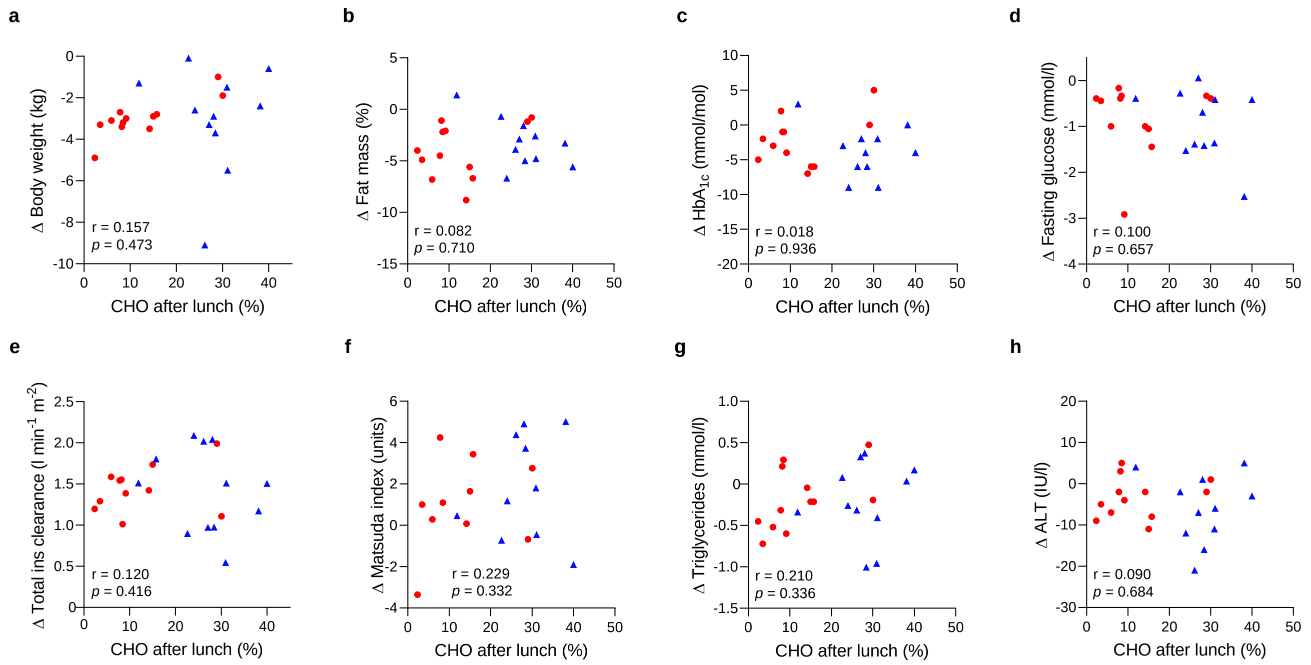


Correlations were tested by Spearman rank correlation.

**ESM Fig. 6** – Changes in body weight (**a**), fat mass (**b**), $HbA_{1c}$ (**c**), fasting glucose (**d**), insulin clearance (**e**), Matsuda index (**f**), triglycerides (**g**), and ALT (**h**) in the first tertile (light blue circles) and third tertile (dark blue squares) of the distribution of percent carbohydrate intake after lunch in individuals with type 2 diabetes randomly assigned to a 12-week eTRC diet or a Mediterranean-style control diet.

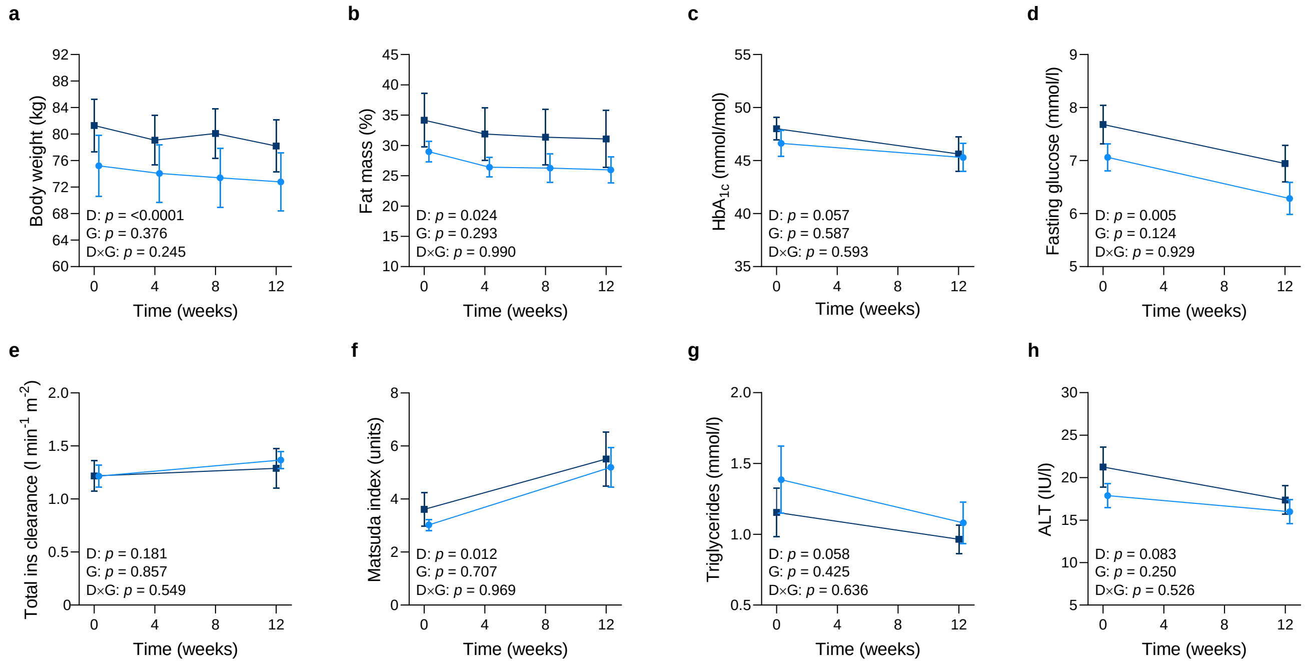


Data are mean ± SEM. Group differences were tested by two-way ANOVA for repeated measures including diet (D), group (G), and diet × group (D×G) interaction as factors.
